# All-trans retinoic acid synergizes with topotecan to suppress AML cells via promoting RARα-mediated DNA damage

**DOI:** 10.1186/s12885-015-2010-6

**Published:** 2016-01-05

**Authors:** Zhifei Xu, JinJin Shao, Lin Li, Xueming Peng, Min Chen, Guanqun Li, Hao Yan, Bo Yang, Peihua Luo, Qiaojun He

**Affiliations:** Zhejiang Province Key Laboratory of Anti-Cancer Drug Research, Institute of Pharmacology and Toxicology, College of Pharmaceutical Sciences, Zhejiang University, 866 Yuhangtang Road, Zijingang Campus, Hangzhou, 310058 Zhejiang People’s Republic of China

**Keywords:** AML, Topotecan, All-trans retinoic acid, Apoptosis, DNA damage, RARα

## Abstract

**Background:**

Chemotherapy is the only therapy option for the majority of AML patients, however, there are several limitations for this treatment. Our aim was to find a new chemotherapy strategy that is more effective and less toxic.

**Methods:**

MTT assays and a xenograft mouse model were employed to evaluate the synergistic activity of all-trans retinoic acid (ATRA) combined with topotecan (TPT). Drug-induced DNA damage and apoptosis were determined by flow cytometry analysis with PI and DAPI staining, the comet assay and Western blots. Short hairpin RNA (shRNA) and a RARα plasmid were used to determine whether RARα expression influenced DNA damage and apoptosis.

**Results:**

We found that ATRA exhibited synergistic activity in combination with Topotecan in AML cells, and the enhanced apoptosis induced by Topotecan plus ATRA resulted from caspase pathway activation. Mechanistically, ATRA dramatically down regulated RARα protein levels and led to more DNA damage and ultimately resulted in the synergism of these two agents. In addition, the increased antitumor efficacy of Topotecan combined with ATRA was further validated in the HL60 xenograft mouse model.

**Conclusions:**

Our data demonstrated, for the first time, that the combination of TPT and ATRA showed potential benefits in AML, providing a novel insight into clinical treatment strategies.

**Electronic supplementary material:**

The online version of this article (doi:10.1186/s12885-015-2010-6) contains supplementary material, which is available to authorized users.

## Background

Acute myeloid leukemia (AML) is the most common acute leukemia worldwide and has high mortality rates, the overall median survival for patients with AML is 6.5 months due to limited treatment choices [1, 2]. The current treatment options for patients with AML are bone marrow transplants, radiofrequency ablation and chemotherapy [3]. Bone marrow transplants are only curative for a small percentage of matching patients [4]. Meanwhile, the efficacy or response rate of radiofrequency ablation in advanced AML is pretty low [5]. Chemotherapy, the only or necessary choice for most AML patients, is often limited by toxicities [6]. Therefore, it is critical to discover effective drug treatments for AML patients.

Topotecan [10-hydroxy-9-dimethylaminomethyl-(S)-camptothecin] (TPT), a semisynthetic topoisomerase 1 inhibitor derived from camptothecin (CPT), is active in patients with different types of solid tumors [7–10]. TPT forms a covalent complex between Topoisomerase 1 and DNA, also called the cleavage complex, resulting in DNA damage during cell replication and transcription, ultimately leading to apoptosis [11, 12]. This mechanism is being investigated for salvage and front-line therapy in AML patients combined with other medicines, such as etoposide, cytarabine, and cyclophosphamide [13–17]. Unfortunately, clinical studies have shown that the activity of these therapies in AML is also limited due to their dose-dependent toxicity. Therefore, discovering potential drugs synergistic to the anticancer activity of TPT that do not enhance its toxicity is critical.

All-trans retinoic acid (ATRA) is a derivative of vitamin A that has good efficacy and has shown less side effects in APL during clinical observations [18]. Experimental studies showed that ATRA treatment in AML affects leukemic cell morphology, regulation of cell cycle progression and apoptosis by activating nuclear receptors, including retinoic acid receptors (RAR types α, β, γ) and retinoid X receptors (RXR types α, β, γ) [19]. Therefore, ATRA is the most promising agent to enhance the antitumor activity of TPT in AML.

In this study, we identified that the combination of TPT with ATRA have synergistic effects to AML *in vitro* and *in vivo*. Our study provides molecular insights for apoptosis involving TPT and ATRA by demonstrating that ATRA helps TPT cause serious DNA damage leading the AML cells HL60 to apoptosis, and RARα is involved in this process. Our results indicate that the TPT-ATRA combination may be a promising alternative chemotherapeutic strategy for AML.

## Methods

### Reagents

ATRA (cat # R2625) was obtained from Sigma (St Louis, MO, USA) and stored in ethanol at −40 °C. TPT, with more than 99 % purity, is synthesized by professor Wei Lu (East China Normal University) and dissolved in dimethylsulfoxide (DMSO) as stock solution at 10 mM. The stock solution was kept frozen in aliquot at −40 °C and thawed immediately prior to each experiment.

### Cell line and cell culture

HL60, NB4 and U937, the human acute myelocytic leukemia cell lines, were obtained from ATCC, and they were maintained in complete media (RPMI-1640 medium (Gibco, Grand Island, NY, USA) supplemented with 10 % heat-inactivated fetal bovine serum (FBS) plus penicillin (100 units/ml) and streptomycin (100 ug/ml)) at 37 °C in 5 % CO_2_.

### Cell Proliferation assay *in vitro*

The cytotoxic activity was detected by MTT assay. After Cells were cultured in 96-well plates at 4 × 10^3^ cells/well, and allowed to proliferate to confluence in 5 % CO_2_ incubator at 37 °C overnight; exposed to different concentrations of TPT, ATRA or TPT combined with ATRA for 48 h. Cells were then incubated with MTT 5 mg/ml (Sigma, USA) for 4 h. A quantity of 100 ml of DMSO (dimethyl sulfoxide) was added to each well after removing the supernatant. Cell viability was obtained by measuring the absorbance on a Multiskan Spectrum (Thermo Electron Corporation, Marietta, Ohio) at 570 nm.

The growth inhibition was calculated according to the following formula: the Growth Inhibition Ratio (IR%) = [(the absorbance of blank control group – the absorbance of experimental group)/the absorbance of blank control group] × 100 %.

### PI staining for flow cytometry

The sub-G1 analysis after PI staining was employed to assess the apoptosis. HL60 cells (10^5^/ml) were seeded into 6-well plates and exposed to either or both of TPT and ATRA for 48 h. Cells were then harvested and washed with ice-cold PBS, fixed with precooled 75 % ethanol at −20 °C overnight. Cells were washed, and resuspended in 500 μl of PBS containing 100 μg/ml RNase (Amersco, Solon, OH, USA), then incubated at 37 °C for 30 min. After incubation, the cells were stained with 200 μg/ml propidium iodide (PI, Sigma, St Louis, MO, USA) in the dark at room temperature for 30 min. For each sample, at least 2 × 10^4^ cells should be analyzed using an FACS-Calibur cytometer (BD Biosciences, Sanjose, CA), and the data were analyzed using cellquest software (BD biosciences).

### JC-1 stain for mitochondrial membrane potential (△Ψm)

HL60 cells were inoculated into 6-well plates (150,000/well) for 24 h growth. After stabilisation for 24 h HL60 were treated with TPT, ATRA or both for 24 h and then harvested. Wash twice with PBS and suspend in 500 μl PBS with 2.5 μl JC-1 (20 μg/ml). Keep the samples in 37 °C, 5 % CO2, for 30 min. Then analyze immediatly with the flow cytometer, typically equipped with a 488 nm argon laser. JC-1 is a cationic dye that exhibits potential-dependent accumulation in mitochondria, indicated by a fluorescence emission shift from green (525 ± 10 nm) to red (610 ± 10 nm). Samples (1 × 104 cells/sample) were analyzed by FACS Calibur (Becton Dickinson, CA, USA).

### Immunofluorescence

For morphological studies, exponentially growing cells were cultured in 3 × 10^5^/well in 6-well plates and treated with TPT, ATRA or both for 48 h. The cells were washed with PBS, fixed with 0.1 % Triton X-100 for 15 min at room temperature, stained with 4’,6-dianidino-2-phenylindole dihydrochloride (DAPI, 2.0 μg/ml, Sigma) for another 15 min. Morphological changes of cell nucleus were examined in a fluorescence microscope, and they were photographed using a digital color camera DFC 300 FX (Leica, Wetzlar, Germany).

### Western blot analysis

The protein samples were prepared as described previously. Briefly, proteins of HL60 cells were extracted in lysis buffer (150.0 mM NaCl, 50 mM Tris–HCl, 1 mM EDTA, 0.1 % SDS, 0.5 % dexoycholic acid, 0.02 % sodium azide, 1 % NP-40, 2.0 μg/ml aprotinin, 1 mM phenylmethylsulfonylfluoride). The lysates were centrifuged at 10^4^ × g for 15 min at 4 °C. Equivalent amounts of proteins were analyzed by 8 %-15 % SDS-PAGE and electroblotted onto PVDF membranes (Millipore Corporation, Billerica, Massachusetts), and probed with primary antibodies. Appropriate antibodies to anti-caspase-3, anti-poly-ADP-ribose polymerase (PARP), anti-Bax, anti-chk1, anti-chk2 and anti-β-actin from Santa Cruz Biotechnology (Santa Cruz, CA, USA); anti-Bcl-2, anti-p-chk1, anti-p-chk2, and anti-γ-H2AX from Cell Signaling Technology (Beverly, MA, USA); and anti-Cytochrome C from Cell Signaling Technology (Boston, MA, USA) were used. The proteins were visualized with peroxidase-coupled secondary antibodies (Southern Biotech, Birmingham, UK), and using the enhanced chemiluminescence detection system (Biological Industries, Beit Haemek, Israel) for detection.

### Alkaline comet assay

The alkaline comet assay, also called alkaline single-cell gel electrophoresis assay, was done according to the procedure of Huang et al. [20]. with minor modification. Briefly, drug-treated HL60 cells (10^5^/ml) were pelleted and resuspended in ice-cold PBS. A 50 μl sample of resuspended cells was the mixed with an equal volume of prewarmed 1 % low-melting point agarose. The cell-agarose mixture was placed on a slide precoated with 0.5 % agarose and spread gently with a coverslip. After 10 min at 4 °C, immersed the slides in precooled lysis buffer [2.5 M NaCl, 100 mM Na_2_EDTA, and 10 mM Tris–HCl (pH 10)] for 90 min in the dark. After soaking with electrophoresis buffer (0.3 M NaOH and 1 mM EDTA) for 20 min, the slides were subjected to electrophoresis for 15 min. The cells were stained with DAPI at last, and individual cells were observed in a fluorescence microscope, and photographed by DFC 300 FX (Leica, Wetzlar, Germany).

### Retroviral infection

To prepare the retroviruses, 293FT cells were plated at 6–8 × 10^5^ cells per well in 6-well plates coated with 20 μg ml^−1^ of poly-ornithine. Twenty-four hours after plating, the cells were transfected with RARα shRNA plasmid, along with pUMVC and pCMV-VSV-G plasmids at the ratio of 2.125 μg of plasmid DNA to 5 μl of Lipofectamine 2000 in 500 μl Opti-MEM (both from Invitrogen). The ratio of shRNA:Pumvc: pCMV-VSV-G was 8:8:1. Twenty-four hours after transfection, the DMEM media was replaced with fibroblast (MEME) media. The next day the culture media containing the retroviruses was harvested and mixed 1:1 with 0.6 μg ml^−1^ polybrene (Sigma-Aldrich). The cell debris in the mixtures was removed using 0.45 μm low-protein-binding filters (Nalgene). The HL60 cells were plated at 2.5 × 10^5^ cells per well in 6-well plates one day before infection with the retroviruses. The retrovirus solution was mixed with the cells for 6 h, then certain amount of fresh 1640 media was added for reducing the toxcity of polybrene. After infection of 24 h, cells were immediately transferred to complete RPMI-1640 supplemented with 10 % fetal bovine serum and cultured at 37 °C until analysis.

### Lentivirus transduction

With Lipofectamine 2000, vesicular stomatitis virus-pseudo-typed vectors were produced by transient cotransfection of 293FT cells with 10 μg of the expression construct (pccl, or pccl-RARa), 10 μg of the pR△8.9 packaging plasmid, and 2 μg of the pMD.G envelope plasmid. Sodium pyruvate (Invitrogen) induction was performed according to the manufacturer’s instructions. After 72 h, the viral supernatants were harvested, centrifuged (800 g, 15 min), and filtered. After determination of the titer of the vector supernatants, HL60 cells were transduced with the indicated lentivirus particles (multiplicity of infection of 1 : 1) in the presence of 8 μgmL^−1^ polybrene. A second cycle of transduction was performed 24 h later with new viral supernatants in the presence of fresh polybrene. After 24 h, the cells were washed and cultured in fresh medium.

### Animals and antitumor activity *in vivo*

Male immune-deficient nude mice at 4 weeks of age (National Rodent Laboratory Animal Resource, Shanghai Branch, China) were maintained in pathogen-free conditions with irradiated chow. Animals were unilaterally, subcutaneously (s.c.) injected with 5 × 10^6^ HL60 cells/tumor in 0.1 ml Matrigel (Collaborative Biomedical Products, Bedford, MA). When HL60 cells formed palpable tumors, mice were divided randomly into 4 groups receiving control (n = 3), TPT alone (n = 3), ATRA alone (n = 3) or combination of both TPT and ATRA (n = 3). ATRA (5 mg/kg) was dissolved in corn oil and given to mice by gavage and TPT (2 mg/kg) was given to mice by intraperitoneal injection every two days of the experimental period. Control group received vehicle (0.5 % methylcellulose, 0.2 % Tween 80 and 99.3 % DDW, i.g. administration) everyday and 0.9 % saline solution by intraperitoneal injection every two days of the experimental period. Body weight and tumors were measured every two days. Tumor sizes were calculated by the formula: (length × width × width)/2, and the length, width and height is in millimeters. At the end of the experiment, animals were sacrificed by CO_2_ asphyxiation and tumor weights were measured after their careful resection. Tumor tissue was collected for analysis. The individual relative tumor volume (RTV) was calculated according to the following formula: RTV = V_N_/V_0_, where V_N_ is the tumor volume on day n and V_0_ is the tumor volume on day of initial treatment. Therapeutic effects of treatment were expressed in terms of T/C% using the calculation formula T/C (%) = mean RTV of the treated group/mean RTV of the control group × 100 % [21]. All procedures were in accordance with the ethical standards of, and the protocols were approved by, the Animal Ethical and Welfare Committee (AEWC), Center for Drug Safety Evaluation and Research, Zhejiang University.

### TUNEL assay

To evaluate the apoptotic response in tumor tissue, we applied terminal deoxynucleotidyl transferase (TdT)-mediated dUTP-digoxigenin nick-end labeling (TUNEL) technique, to formalin-fixed tumor samples in paraffin blocks, using the one-step TUNEL apoptosis assay kit produced by Beyotime Institute of Biotechnology in China. The sections (4–5 μm) mounted on glass slides were deparaffinized, rehydrated thoursough graded alcohols to water, treated with 20 μg/ml proteinase K (37 °C, 20 min) and then washed in 1 × Tris buffer. TUNEL assay was then performed according to the manufacturer’s instructions.

### Statistical analysis

Data were presented as mean ± SD for thoursee separate experiments. Comparions between groups were made with unpaired Student’s two-tailed *t* test and *P* < 0.05 was considered statistically significant.

## Results

### TPT and ATRA synergistically induce a cytotoxic effect in AML cells

**Fig. 1:**
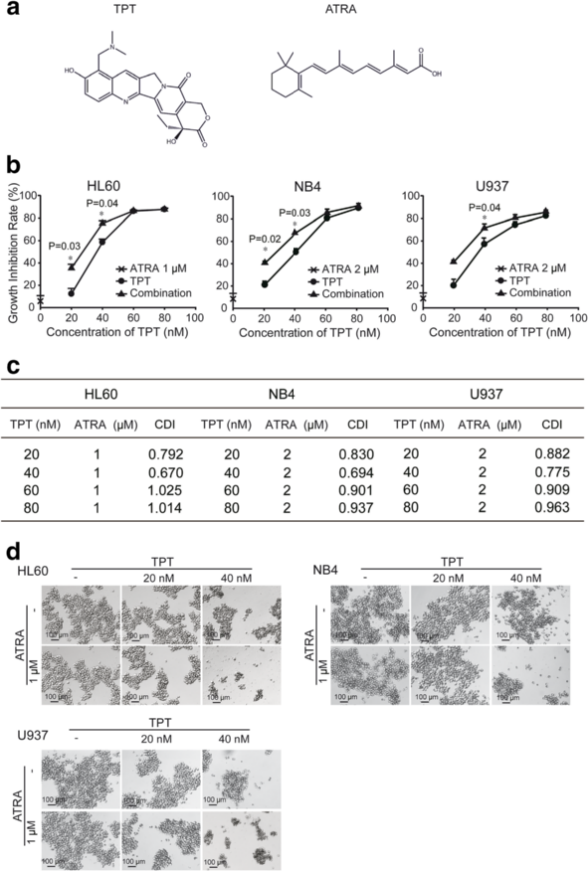
Combinational cytotoxicity of Topotecan (TPT) and ATRA. **a** Chemical structures of TPT and ATRA. **b** Combination of TPT and ATRA induce cytotoxicity in HL60, NB4 and U937 cells. 4 × 103 cells per well were cultured in 96-well plates and incubated with the indicated concentrations of TPT and ATRA for 48 h. Mean ± SD from three independent experiments. *: Compared to TPT group, p < 0.05. **c** CDI values at different ratio concentrations of TPT and ATRA (1 or 2 μM) were calculated by the proliferation inhibition rates. Each study was performed three times and the error bars represent the SD around the mean. **d** Cell morphology change was observed by microscope (magnification 200×) after combination of TPT and ATRA

To evaluate the potential synergy between ATRA and the Topoisomerase1 inhibitor TPT, cells (HL60, NB4 and U937) were exposed for 48 h to increasing concentrations of TPT (0, 20, 40, 60, 80 nM) alone or in combination with ATRA at a concentration that reduces cell viability according to data from preclinical research and our laboratory. As shown in Fig. 1b, a synergistic effect was observed in ATRA combined with lower concentrations of TPT (20 nM and 40 nM). However, as the concentration of TPT increased the cytotoxicity appeared much more severe with no significant differences between the TPT group (60 nM and 80 nM) and the TPT plus ATRA group. To validate the combination efficiency, we calculated the Coefficient of Drug Interaction (CDI) values, which are used to quantify drug synergism. CDI values less than, equal to or greater than 1 indicate that the drugs are synergistic, additive or antagonistic, respectively. CDI values less than 0.7 indicate that the drugs are significantly synergistic. The CDI values for different ratio concentrations of ATRA and TPT were calculated. As shown in Fig. 1c, the combination of ATRA and TPT had apparent synergism in HL60, NB4 and U937 cells with CI values <1. Cells treated with the combination of TPT and ATRA showed cell morphology changes and had more cell debris appearing compared to TPT or ATRA groups in Fig. 1d. Thus, we demonstrated that ATRA has synergistic cytotoxicity with TPT in AML cells.

### ATRA enhances TPT–triggered caspase-dependent apoptosis *in vitro*

**Fig. 2:**
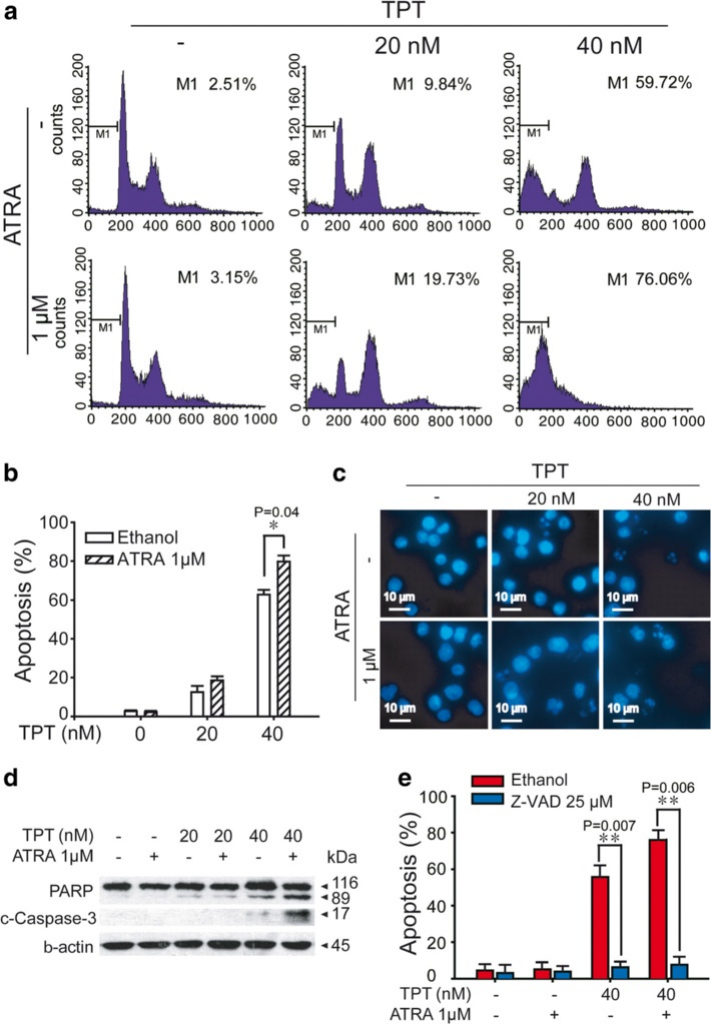
Combination of TPT and ATRA induce apoptosis in HL60 cells. **a** and **b** Effects of ATRA in combination with TPT and ATRA on cell cycle kinetics of HL60 cells. Cell cycle analysis was done after propidium iodide (PI) staining on cells exposed to TPT or ATRA either alone or in combination as indicated. *: Compared to 40 nM TPT group, p < 0.05. **c** Morphology of apoptotic bodies in HL60 cells treated with TPT, ATRA, or combination for 48 h. Cells were stained with DAPI and observed by fluorescence microscope (magnification 200×). **d** HL60 cells were exposed to TPT, ATRA, or combination for 24 h, after which, protein extracts were immunoblotted with specified antibodies for PARP and cleaved-caspase-3. **e**. HL60 cells were pretreated with 25 μM Z-VAD-FMK (the pan-caspase inhibitor) for 1 h and treated with TPT and/or ATRA for 24 h. The cells were analysed on flow cytometry. *: Compared to ethanol group, p < 0.05. The experiments were performed three times independently, and the error bars represent the SD around the mean

To further explore the mechanisms of enhanced antitumor activity caused by the combination of TPT and ATRA, we examined their effects on apoptosis and caspase signaling pathways. Flow cytometry analysis after PI staining was used first to identify the apoptosis-inducing effects. As shown in Fig. 2a, PI staining for sub-G1 content analysis was used to characterize the apoptosis process in HL60 cells treated with TPT (20 nM and 40 nM), 1 μM ATRA or the combination for 48 h. Exposure to ATRA drove a few cells to apoptosis, and the content of sub-G1 was close to the control group (approximately 2 %). However, using ATRA with TPT generated more apoptotic cells compared to TPT treatment alone. Then, DAPI staining further showed that the combination of 1 μM ATRA and TPT for 48 h triggered more apoptosis compared to the treatment of either drug alone. As Fig. 2c depicts, apoptotic bodies were seldom found in control cells and the monotreated ATRA cells. However, apoptotic bodies appeared in cells exposed to TPT. Adding ATRA showed more apoptotic bodies with the accompanied nuclei shrinking and disintegrating.

To explore the signaling mechanism for TPT and ATRA synergistic induction of apoptosis, we investigated the expression levels of apoptosis-related proteins, such as caspase-3 and PARP, as shown in Fig. 2d, using Western blot analysis. Incubation with TPT at the indicated concentrations (20 nM and 40 nM) combined with ATRA (1 μM) for 24 h caused significant cleavage of PARP compared to the monotreated cells. Pro-caspase-3, upstream PARP [22], was cleaved more severely with the combination of 40 nM TPT and ATRA. Then, we used Z-VAD-FMK, the pan-caspase inhibitor, to further confirm the mechanism previously mentioned. As shown in Fig. 2e, the apoptosis induced by the combination treatment was almost reversed in the presence of Z-VAD-FMK, as detected by the PI (sub-G1) staining assay. All of these results indicated that caspase involvement led to apoptosis triggered by TPT plus ATRA. In summary, our results demonstrated that a combination treatment of TPT and ATRA triggers apoptosis via caspase cascades leading to increased cell death.

### Mitochondrial damage was involved in caspase-dependent apoptosis

**Fig. 3:**
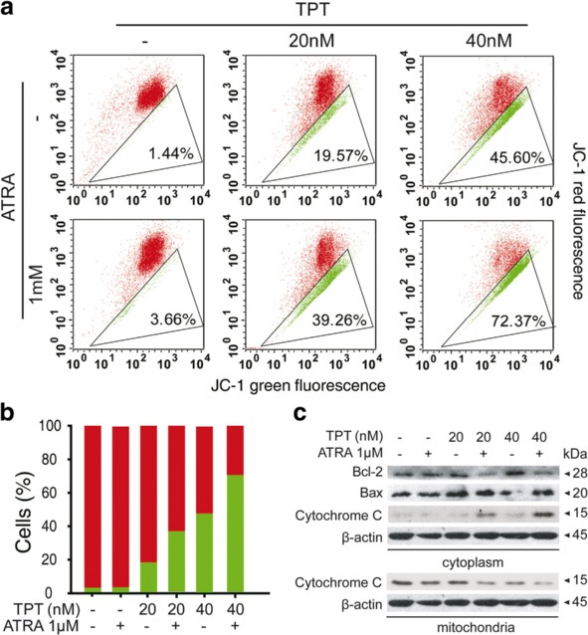
Mitochondrial damage was involved in caspase-dependent apoptosis. **a** and **b** Effects of TPT, ATRA, or combination for 24 h on loss of mitochondrial membrane potential in HL60 cells were detected by flow cytometry after JC-1 staining. **b** The rates of red to green after HL60 Cells treated with TPT, ATRA, or combination for 24 h. **c** Effects of TPT, ATRA, or combination on Bcl-2, Bax and Cytochrome C protein expression were analyzed by western blot

Mitochondrial damage in response to cellular conditions is an important constituent of apoptosis, and the mitochondrial membrane potential is one of the most remarkable events [23]. In order to explore the role of mitochondrial damage in apoptosis induced by a combination treatment of TPT and ATRA, JC-1 staining and flow cytometry were conducted after a 24 h treatment. The results showed that cells treated with the combined TPT and ATRA induced the loss of mitochondrial membrane potential compared to TPT or ATRA groups in Fig. 3a-b. The oncoprotein Bcl-2 is an antagonist of the mitochondrial pathway for apoptosis [24]. BAX is a member of the Bcl-2 gene family that forms a heterodimer with bcl-2 and functions as an apoptotic activator [25]. As shown in Fig. 3c, Western blot analysis revealed that bcl-2 is down-regulated and bax is up-regulated in HL60 cells treated with TPT and ATRA together. Cytochrome C, which normally resides exclusively in the intermembrane space of mitochondria, is released into the cytosol during apoptosis. Cytochrome C release from mitochondria to the cytoplasm was shown to be up-regulated in HL60 cells treated with TPT and ATRA together, as depicted in Fig. 3c. These data suggest that the combination treatment of TPT and ATRA induced mitochondrial associated-apoptosis.

### Induction of apoptosis mediated by the combined treatment of TPT and ATRA is correlated to enhanced DNA damage in AML cells

**Fig. 4:**
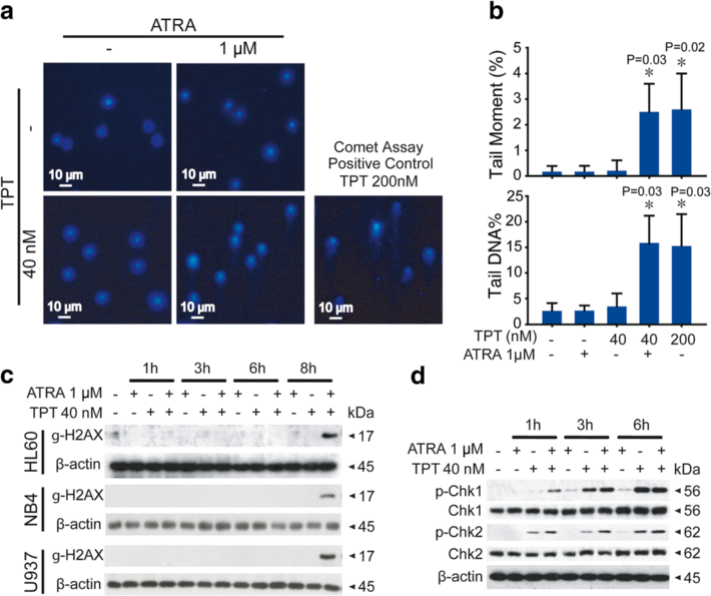
ATRA synergistically induced DNA damage in combination with TPT. **a** and **b** HL60 cells were treated with or without 40 nM TPT or 1 μM ATRA for 1 h. Alkaline comet assay was used for detection of single-strand breaks (SSBs). Representative comet images of each group were shown and 200nM TPT served as the positive control (magnification 200×). Tail Moment and Tail DNA% were analysised the data from Comet assay. Each point represents mean ± SD of three independent experiments. *Significantly different (P < 0.05) from the control, according to the Dunnett’s test. **c** and **d** AML cells were exposed to TPT (40 nM), ATRA (1 μM), or in combination for indicated times, after that protein extracts were immunoblotted with specific antibodies of γ-H2AX (**c**), p-Chk1/2, Chk1/2 (**d**)

TPT is a cytotoxic drug that adheres Topoisomerase 1 to DNA, which inhibits the function of Topoisomerase 1 in DNA repair [12]. Trapped Topoisomerase 1 leads to single strand DNA breakage and then to cell death. We used the comet assay to determine if ATRA plays a role in TPT induced-DNA damage. The alkaline version detects both single-strand and double-strand breaks (DSBs), and the neutral version only detects double-strand breaks [26, 27]. Exposure to different concentrations of TPT and ATRA for one hour showed obvious “comet tails” in two of the groups: the combination group and the positive control group for 200 nM TPT. The other groups showed only obscure “halos” without clear directions (Fig. 4a). The results demonstrated that treatment with ATRA and TPT for one hour induced HL60 DNA damage with 200 nM TPT. However, neither treatments of 1 μM ATRA or 40 nM TPT had similar effects.

Because the “tails” were caused by single-strand or double-strand breaks, we next detected the level of phosphorylated-H2AX (γ-H2AX) in DNA DBSs that are accompanied by the elevation of γ-H2AX. Phosphorylation of histone H2AX on serine 139 was one of the earliest cellular responses after the formation of DNA damage [28]. This phosphorylated form of H2AX (referred to as γ-H2AX) was used as a marker for the presence of DNA damages. AML cells, including HL60, NB4 and U937 cells, were incubated with TPT, ATRA, or both and proteins were collected at 1, 3, 6, and 8 h, respectively. As shown in Fig. 4b, γ-H2AX expression increased from a very low level to a high level, which demonstrated that only exposure to TPT and ATRA for eight hours induced DBSs. Therefore, the “comet tails” formed after one hour of drug treatment were caused by SSBs (Fig. 4c). TPT inhibits topoisomerase1 and causes single-strand DNA breaks, which inhibit DNA function and ultimately lead to cell death by generating double-stranded DNA breaks during DNA replication [29]. Furthermore, we examined p-Chk1 and p-Chk2 which are essential for responses to DNA damage [30]. These two checkpoint kinases were improved with the combination treatment of TPT and ATRA (Fig. 4d). These results indicated that ATRA and TPT synergistically induced apoptosis by causing serious DNA damage.

### RARα was involved in TPT-induced DNA damage

**Fig. 5:**
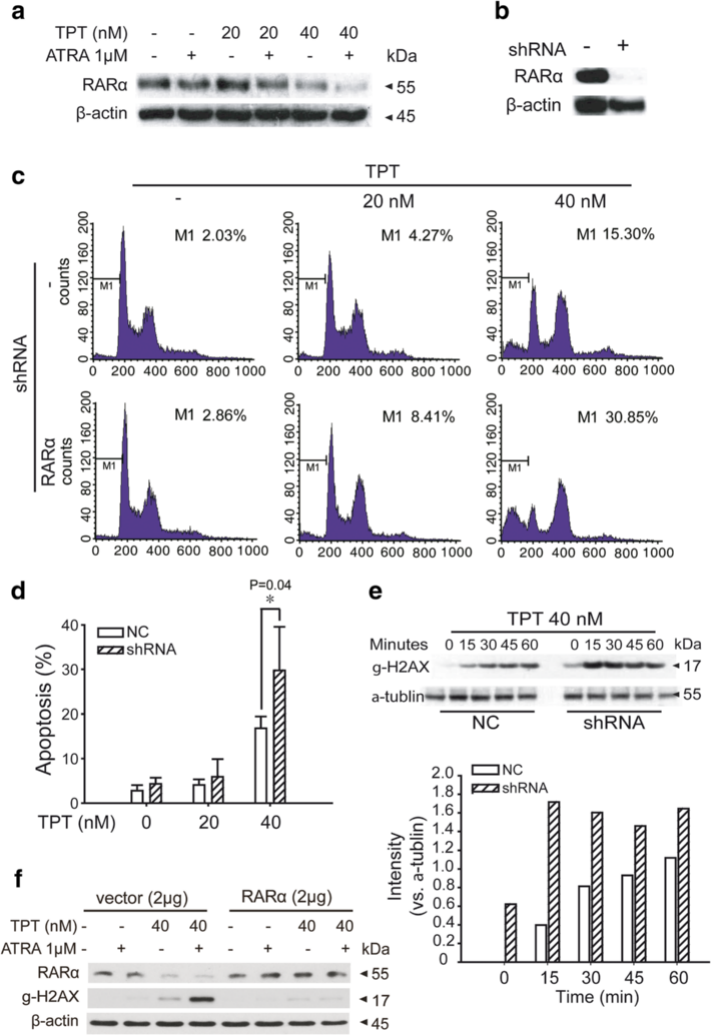
RARα participated in synergistic induction of apoptosis by TPT and ATRA treatment. a After incubation with TPT (40 nM), ATRA (1 μM), or combination for 24 h, the lysates of HL60 cells were prepared for western blot analysis of RARα expression. **b**. HL60 cells were transfected by negative control (NC) or shRNA of RARα. Western blot analysis showed decreased RARα protein levels in both cell lines. **c** and **d** After infection of 72 h, cells were treated with TPT for 24 h. The apoptosis ratio of HL60 cells were detected by flow cytometry. *: Compared to TPT group, p < 0.05. The experiments were performed three times independently, and the error bars represent the SD around the mean. **e** After transfection of NC or siRNA of RARα, HL60 cells were treated with TPT for indicated minutes. Protein expression of γ-H2AX was analyzed by western blot and densitometry quantification based on α-tublin expression. **f** After transfection of vector or RARα plasmid, HL60 cells were treated with TPT for indicated minutes and protein expression of γ-H2AX was analyzed by western blot

RARα, the main target of ATRA, plays an important role in ATRA-therapies. Previous data has shown that ATRA elevates the activity of proteasomes leading to RARα protein degradation by the ubiquitin-proteasome pathway [31, 32]. We found similar results for the ATRA treatment with prolonged time (Fig. 5a). Interestingly, the combination of ATRA and TPT downregulates RARα. To explore the role of RARα in this combination therapy, retroviral shRNA was used to knockdown the RARα expression levels in HL60 cells (Fig. 5b). Two days later, the cells were incubated with TPT for 24 h before PI staining and flow cytometry to detect cell apoptosis. Figure 5c-d shows that treating with TPT for the same amount of time leads to cells with lower expression of RARα and higher apoptosis percentages than in normal cells. From the comparisons between the control group and RARα-knockdown group, we concluded that low levels of RARα proteins are beneficial for TPT-induced apoptosis.

To determine whether RARα influences TPT-induced apoptosis through the DNA damage signaling pathway, we examined the related protein γ-H2AX. Increasing phosphorylation of H2AX was observed in Western blots for cells with low RARα expression in the combination therapy group (Fig. 5e). To further confirm the role of RARα in DNA damage, lentivirus transduction was used to overexpress RARα in HL60 cells. Figure 5f shows that the increasing phosphorylation of H2AX was reversed in the RARα-overexpression group, and this observation demonstrated that the downregulation of RARα may be a key factor for the accumulation of DNA damage and apoptosis.

### Synergistic antitumor efficacy of TPT and ATRA in HL60 xenografts

**Fig. 6:**
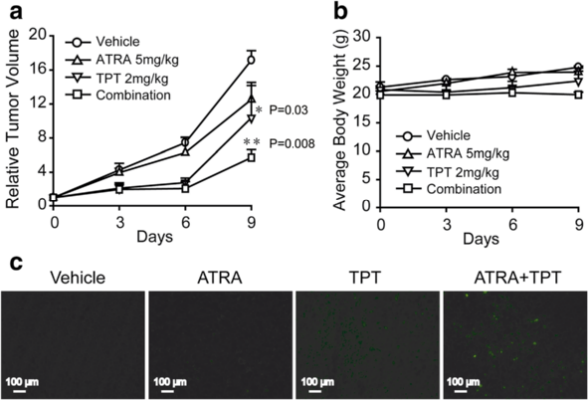
Efficacy of TPT combined with ATRA treatment regimen *in vivo*. Mice transplanted with HL60 human xenografts were randomly divided into 4 groups and given injection of TPT (2 mg/kg, i.p.), ATRA (5 mg/kg, i.g.), combination, or vehicle for a period of 9 days. **a** Relative tumor volume was expressed as mean ± SD (n = 3 per group). **b** The average body weight of each group was expressed as mean ± SD (n = 3 per group). **c** Representative fluorescence images of TUNEL staining of tumor tissues collected from each group (magnification 100x)

In light of the *in vitro* synergistic effect of TPT and ATRA, we studied the *in vivo* anticancer activity of the combination therapy in nude mice bearing HL60 xenografts as described in the Materials and Methods. Figure 6a shows that the i.p. administration of ATRA at a dose of 5 mg/kg twice per week for nine days produced no significant difference in the mean RTV compared to the control group (mean RTV, ATRA vs. control: 12.5 vs. 17.1; P > 0.05). However, after a dosage of 2 mg/kg every week for nine days, TPT exerted a moderate tumor growth inhibitory effect (mean RTV, TPT vs. control: 10.4 vs. 17.1; P < 0.05). As predicted, TPT plus ATRA caused marked tumor growth inhibition (T/C value: 33.3 %) that was significantly greater than TPT (T/C value: 60.8 %) or ATRA treatment alone (T/C value: 73.1 %; mean RTV, combination vs. TPT: 5.7 vs.10.4; P < 0.01). Furthermore, compared to the initial body weights, combination treated mice showed no significant body weight loss in Fig. 6b.

The TUNEL assay was performed to evaluate the apoptosis-inducing abilities of the TPT and/or ATRA treatments. As shown in Fig. 6c, the number of TUNEL-positive cells was significantly increased in the tumor tissues of combination-treated mice. All *in vivo* data were consistent with previous *in vitro* data and further supported that the synergistic antitumor efficacy of TPT and ATRA was a result of TPT aroused apoptosis.

## Discussion

Acute myeloid leukemia is most often diagnosed in older people and children; more than 50 % of patients with AML are over-60 and 15-20 % are under 16 years old [33, 34]. Chemotherapy is the only treatment option for the majority of AML patients and the most frequently used drugs are the deoxycytidine analog cytarabine and an anthoursacycline antibiotic, such as daunorubicin, idarubicin and the anthoursacenedione mitoxantrone [3]. However, multiple chemotherapy treatments are intolerable for children and older people with AML, therefore, new effective therapies with fewer side effects are urgently needed. In this study, we demonstrated that ATRA had a synergistic cytotoxicity with TPT for AML *in vitro* and *in vivo* closely related to DNA damage-induced apoptosis via RARa activity inhibition.

ATRA used in combination with chemotherapy has been shown to improve the outcome of patients with breast cancer, lung cancer, ovarian cancer and gastric cancer, and only presents a few side effects, which suggests a potential for clinical application in AML [35, 36]. Previous studies in ovarian, gastric and melanoma cancer cells have shown that retinoic acid has synergistic effects on DNA damage with the drug cisplatin [37]. TPT is effective alone with cytotoxicity effects less than the doxorubicin (a classical AML drug) group (Additional file 1: Figure S1) or when combined with other drugs for AML, such as lapatinib, paclitaxel. However, TPT is limited by its toxicity [14, 16, 17]. ATRA was proposed as a potential drug to enhance the anticancer activity of TPT. We demonstrated that ATRA decreases the concentration that causes DNA damage from 200 nM to 40 nM TPT.

DNA integrity is critical for proper cellular function and proliferation in AML. Once a DNA lesion occurs, it leads to replication-associated DNA double-strand breaks (DSBs) that eventually cause apoptosis if the damaged DNA cannot be properly repaired [38]. Targeted therapies designed to induce apoptosis in leukemic cells are currently the most promising antileukemia strategies. We used flow cytometry analysis with PI staining, morphological evidence of apoptotic bodies, and immunoblotting to determine if the ratio of growth inhibition was induced by caspase-mediated apoptosis. The comet assay revealed that treatment with TPT and ATRA for one hour induces DNA SSBs in HL60 cells at high concentrations of TPT, although neither 40 nM TPT or 1 μM ATRA caused the SSBs first when using ATRA combined with TPT for one hour. Moreover, the DNA damage pathway and its transducers (Chk1, Chk2) were activated by TPT and ATRA at higher rates than TPT. Over time, the SSBs became DSBs and finally led to cell death by apoptosis.

ATRA binds to the retinoic acid receptor (RAR), RAR-alpha, RAR-beta, and RAR-gamma, which is bound to DNA as a heterodimer with the retinoid X receptor (RXR) in regions called retinoic acid response elements (RAREs), from there it affects gene transcription and modulates a wide variety of biological processes, such as apoptosis [31]. In our study, RARα was knocked-down and the cells were susceptible to TPT, which increased the apoptosis rate. Therefore, we concluded that the existence of RARα was unfavorable for programmed cell death. The combination regime showed that ATRA downregulates the protein level of RARα, and this might be the key for synergistic effects in this anticancer therapy. As a result, DNA damage can be induced more easily with ATRA. However, the influence of RARα on TPT-induced DNA damage has not been explained, and the role of ATRA in the DNA damage pathway needs further exploration.

## Conclusion

In summary, the combination of TPT and ATRA exhibited synergistic antitumor activity *in vitro* and *in vivo* demonstrated by a decreased tumor cell survival fraction, increased inhibition of tumor cell proliferation, significant activation of apoptosis cascades and an increased tumor growth inhibitory rate. Moreover, the synergistic activity was related to the suppression of RARα proteins and caused single-strand DNA breaks, ultimately leading to cell death by generating double-stranded DNA breaks during DNA replication. Our results indicate that the combination therapy is a promising alternative to chemotherapeutic treatment for AML and should be further tested in clinical trials.

## Additional file

Additional file 1: Figure S1. The TPT effect compared with doxorubicin (ADR). TPT induced cytotoxicity was Compared with ADR in AML cells. 4 × 103 cells per well were cultured in 96-well plates and incubated with the indicated concentrations of TPT and ADR for 48 h. Mean ± SD from three independent experiments. (TIF 10371 kb)
