# Supplementary figures and images for: All-trans retinoic acid synergizes with topotecan to suppress AML cells via promoting RARα-mediated DNA damage
Source: BMC Cancer. 2016 Jan 5;16:2. doi: 10.1186/s12885-015-2010-6 (PMC4700651; doi:10.1186/s12885-015-2010-6)

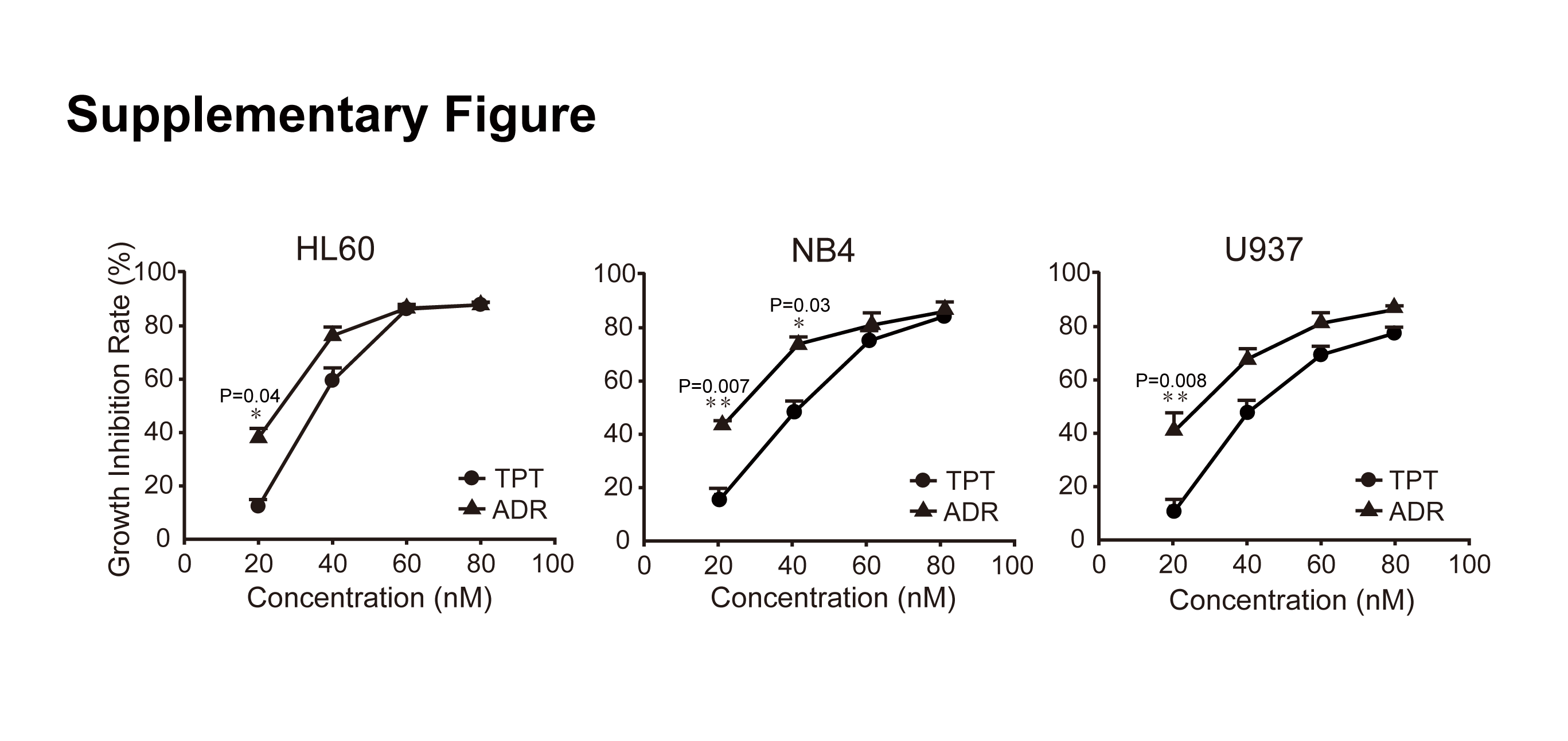

Supplement: Additional file 1: Figure S1. — The TPT effect compared with doxorubicin (ADR). TPT induced cytotoxicity was Compared with ADR in AML cells. 4 × 10^3^ cells per well were cultured in 96-well plates and incubated with the indicated concentrations of TPT and ADR for 48 h. Mean ± SD from three independent experiments. (TIF 10371 kb) [file 12885_2015_2010_MOESM1_ESM.tif]
